# Yuan Fen 緣份

**DOI:** 10.1200/GO.22.00110

**Published:** 2022-05-20

**Authors:** Annie N.M. Wong

**Affiliations:** ^1^ Wellington Blood & Cancer Centre, Wellington Regional Hospital, Wellington, New Zealand; ^2^ University of Otago, Wellington School of Medicine, Wellington, New Zealand

The thing about being invisible is that it is a disability and a gift. Many of us immigrant doctors share a coming-of-age story where we find that what marks us out as different, and what might once have been a source of shame, also grants us access to things that are unreachable to others. We immigrant children spend our formative years trying to wish our differences away, shedding our accents and keeping a low profile. We code-switch so that we can hide the things we eat or the way we dress and the way we talk so as to keep any excuse for taunting to a minimum. Code-switching is a term that originates from linguistics but is used more commonly in the literature about race or sexuality. Code-switching involves members of a minority group adjusting their speech or behavior so to blend into the mainstream culture or workplace. But we learn later that our differences can not only serve other members of the immigrant community but also open up a shared humanity that is equally as rewarding for ourselves.

For me as an immigrant from Hong Kong living in New Zealand, I especially feel my worth when I look after Chinese patients. Usually, these patients are older and their English is limited, so I often see a look of relief when they see their doctor is someone who looks like them. Nothing pleases me more than being able to speak to them in their native tongue. The tales are usually of hardship, perseverance, and hope. They relish the ability to tell their own story, without their family interpreting or filtering, and without using a translator who would often be from the same tight-knit community.

Part of the pleasure for me is the act of speaking in Chinese, which was once a shameful flag to my otherness and something to hide and only to be spoken at home. Now, I can use the language professionally and help my patients get the care they need. My Chinese patients and I establish an immediate bond, and they become more akin to family. We also learn so much about someone by the words they use and the way they speak. We as health professionals know the power of hearing someone's history in a way that no diagnostic scan can replace or the understanding of one's values that is crucial in shared treatment decision making. When we treat immigrants, sometimes what can be lost in translation is the patient's sense of humor or that slight quiver in the end of the sentence that opens up those deep and vulnerable conversations we share in oncology.

One story that encompasses all these things revolves around Mr L. I probably saw my father in him. After all, they were a similar age. Both he and my father loved roast pork glistening with fat and mooncakes stuffed with sweet lotus paste with salted egg yolks. Both were hassled by their wives for neglecting their health. Mr L. attended the hospital for an unrelated problem and was discharged with the diagnosis of metastatic cholangiocarcinoma. Because he felt so well, it was hard for him and the family to really believe that he had a terminal diagnosis. I learned that he left the southern provinces of China with the shirt on his back and worked through relatives' kitchens until he could pinch and save enough for a small shop. Initially, his shop mainly catered to other Chinese people looking for foodstuffs that reminded them of home. But the sauces and pickles soon became a booming business that fed his whole family. He worked hard so that his children need not join the business and instead became professionals, which made him proud. They were destined for more security and happiness than what he himself could afford.

We talked openly about his diagnosis and prognosis. Unlike some Asian families, there were no secrets and we all shared the medical information. Yet, he shared secrets with me when he escaped his relatives and came alone to his oncology appointments. He confided that he “snuck out… they wanted me to try this diet and that diet and all I want is to crunch the crispy crackling…” We talked a lot about foods he was missing, and we talked about his worries for his children, whether they would meet someone, and whether they would settle down. Now that he has passed, when I meet some of his relatives, I only wish that Mr L. too could have met them and seen how things have worked out.

For me, looking after other immigrants is more than just providing medical care. It is also a window to values from our shared culture. Mr L. was always at peace as he saw himself as part of ribbon of life connected to his family, and his own health is only a thread in this woven rope to the future generation. In medical school, we learned about autonomy and individualism as a pillar of medical ethics. In clinic, Mr L. reminded me that health is not only pertaining to the individual, instead, as is true for many Chinese families, the health of the family ecosystem is far more important. For immigrant children, we spend a long time trying to conceal ourselves and parts of our identities, so it is easy to feel we no longer have a firm connection to our roots. It turns out that we were never invisible, as hard as we may try to camouflage, and that our diversity is actually a way that we can contribute to our medical community.

There is this word 緣份 *yuan fen* in Chinese, which does not have a direct translation in English. It is a type of fate that brings people together. The saying goes that if you are fated together, then you will meet even when you are thousands of miles apart; without this fate, you could be face-to-face and not know each other. Knowing these patients and trusting each other to laugh and cry together is such a privilege. Perhaps it is one immigrant seeing another and knowing the feeling of being cast out into the world and what it is like to finally meet someone who can see you exactly as you are.

